# Periodontal Disease and Risk of Head and Neck Cancer: A Meta-Analysis of Observational Studies

**DOI:** 10.1371/journal.pone.0079017

**Published:** 2013-10-23

**Authors:** Xian-Tao Zeng, Ai-Ping Deng, Cheng Li, Ling-Yun Xia, Yu-Ming Niu, Wei-Dong Leng

**Affiliations:** 1 Department of Stomatology, Taihe Hospital and School of Stomatology, Hubei University of Medicine, Shiyan, Hubei Province, People’s Republic of China; 2 Department of Neurosurgery, Taihe Hospital, Hubei University of Medicine, Shiyan, Hubei Province, People’s Republic of China; 3 Department of Oral and Maxillofacial Surgery, School and Hospital of Stomatology, Wuhan University, Wuhan, Hubei Province, People’s Republic of China; Johns Hopkins University, United States of America

## Abstract

**Background:**

Many epidemiological studies have found a positive association of periodontal disease (PD) with risk of head and neck cancer (HNC), but the findings are varied or even contradictory. In this work, we performed a meta-analysis to ascertain the relationship between PD and HNC risk.

**Methods:**

We searched the PubMed, Embase, and Cochrane Library databases for relevant observational studies on the association between PD and HNC risk published up to March 23, 2013. Data from the included studies were extracted and analyzed independently by two authors. Meta-analysis was performed using RevMan 5.2 software.

**Results:**

We obtained seven observational studies involving two cohort and six case-control studies. Random-effects meta-analysis indicated a significant association between PD and HNC risk (odds ratio = 2.63, 95% confidence interval = 1.1.68 - 4.14; *p* < 0.001), with sensitivity analysis showing that the result was robust. Subgroup analyses based on adjustment for covariates, study design, PD assessment, tumor site, and ethnicity also revealed a significant association.

**Conclusions:**

Based on currently evidence, PD is probably a significant and independent risk factor of HNC.

## Introduction

Head and neck cancer (HNC) is the tenth most common cancer worldwide and the seventh most common cause of cancer death [1]. HNC mainly originates in the oral cavity, pharynx, and larynx. Squamous cell carcinomas (SCCs) account for 90% of HNC. Based on 2008 cancer statistics, SCC of the head and neck (HNSCC) has an estimated incidence of 35310 new cases and an expected 7590 deaths in the USA [2]. Therefore, risk factors of HNC must be determined to guide risk -reduction interventions, prevention therapy, and lifestyle changes. Over the past decades, numerous epidemiological studies have identified smoking and alcohol as the major risk factors of HNC [3], other risk factors include genetic factors, viral infection (mostly human papillomavirus), sex, and occupational exposure [1,3]. However, these markers do not comprehensively explain the etiology of HNC.

Periodontal disease (PD) is a group of inflammatory diseases affecting the supporting tissues of teeth. PD affects at least 35% dentate adults aged 30 to 90 years in the USA [4], and up to 90% of the worldwide population [5]. Based on the “focal infection” theory that emerged at the beginning of the 20th century, many studies over the past two decades have investigated the possibility of PD being a risk factor of systemic conditions [6], such cardiovascular diseases [7], diabetes [8], chronic obstructive pulmonary diseases [9], adverse pregnancy outcomes [10], etc. 

In 1863, Virchow [11] hypothesized that cancer originates from chronic inflammation sites, and subsequent evidence has indicated that inflammation is a critical component of cancer progression. Given that PD is a group of inflammatory diseases and that the anatomic relationships exist between PD and HNC, the possible association between these diseases has gained the interest of researchers. In 2005, Tezal et al [12] suggested that PD is associated with increased risk of oral tumors (non-specific). Later epidemiological studies have investigated the link between PD and risk of HNC, and most of them have found a positive association. However, some of the results are varied or even contradictory [12–18]. Therefore, the association between PD and HNC is still unclear.

In this study, we performed a meta-analysis of eligible observational studies to more precisely estimate the association between PD and risk of HNC. This study was reported following the Preferred Reporting Items for Systematic Reviews and Meta-Analyses (PRISMA) statement [19] and Meta-analysis of Observational Studies in Epidemiology (MOOSE) guidelines [20].

## Methods

### Literature search

We searched the PubMed, Embase, and Cochrane Library databases for studies that investigated the association between PD and HNC published up to March 22, 2013. The following search terms were used: (1) "head and neck cancer" OR "oral cancer" OR "oropharyngeal cancer" OR "pharyngeal cancer" OR "laryngeal cancer" and (2) "periodontal disease" OR "periodontitis" OR "periodontal attachment loss" OR "periodontal pocket" OR "alveolar bone loss". We also reviewed the reference lists of pertinent articles and recent reviews.

### Eligibility criteria

Cohort studies, case-control studies, and cross-sectional studies evaluating the risk of HNC events in relation to PD were considered eligible for inclusion if the following criteria were met: (1) full-text could obtain; (2) clear diagnostic criteria for PD and HNC were reported; and (3) the adjusted and/or unadjusted hazard ratios (HRs), odds ratios (ORs) or relative risks (RRs), and associated 95% confidence intervals (CIs) or the numbers of events that can calculate them were reported. If more than one study covered the same population, only the report containing the most comprehensive information on that population was included. Two authors independently evaluated the eligibility of all retrieved studies, and disagreements were resolved by discussion or consultation with a third author.

### Data extraction

Two authors independently extracted and tabulated the following basic information on each study: first author’s surname, publication year, design, country of origin, sample size, number of events, age range, assessment of PD and HNC, tumor site and pathologic type of HNC, crude and/or adjusted point estimates and 95% CIs, and covariate features included in the multivariable model.

### Data analysis

ORs were used to measure association across studies. HRs were directly considered as RRs, and RRs were transformed into ORs with this formula [21]:*RR*=*OR*/[(1−*P*_0_)+(*P*_0_×*OR*)], where *P*_0_ is the incidence of the outcome of interest in the non-exposed group.

The standard error (SE) of the resulting converted OR was subsequently determined using the formula: *SElog*(*RR*)=*SElog*(*OR*)×*log*(*RR*)/*log*(*OR*). Given that these transformations can overestimate the variance of ORs derived from RRs [22], we performed a sensitivity analysis by omitting the study in which this transformation had been applied.

We computed pooled ORs and relevant 95% CIs using Review Manager (RevMan, version 5.2, Copenhagen: The Nordic Cochrane Centre, The Cochrane Collaboration, 2012). We transformed ORs by taking their natural logarithms as well as calculating SEs and the corresponding CIs [23]. We then pooled log (ORs) and their SEs using the inverse variance method. Heterogeneity was assessed with the *I* ^2^ statistic [24] with low, moderate, and high *I* ^2^ values of 25%, 50%, and 75%, respectively [25]. When *I* ^2^ ≤ 25% (indicating no evidence of heterogeneity), we used the fixed effects model; otherwise, the random-effects model was used. We generated forest plots sorted by the publication year.

When heterogeneity was present, we performed a sensitivity analysis excluding studies reporting only HRs or RRs. Planned subgroup analyses were conducted based on the geographical region, study design, PD assessment method, tumor sites, and crude and adjusted ORs. Finally, we assessed evidence of publication bias by visually inspecting of funnel plot.

## Results

### Study selection and characteristics

**Figure 1:**
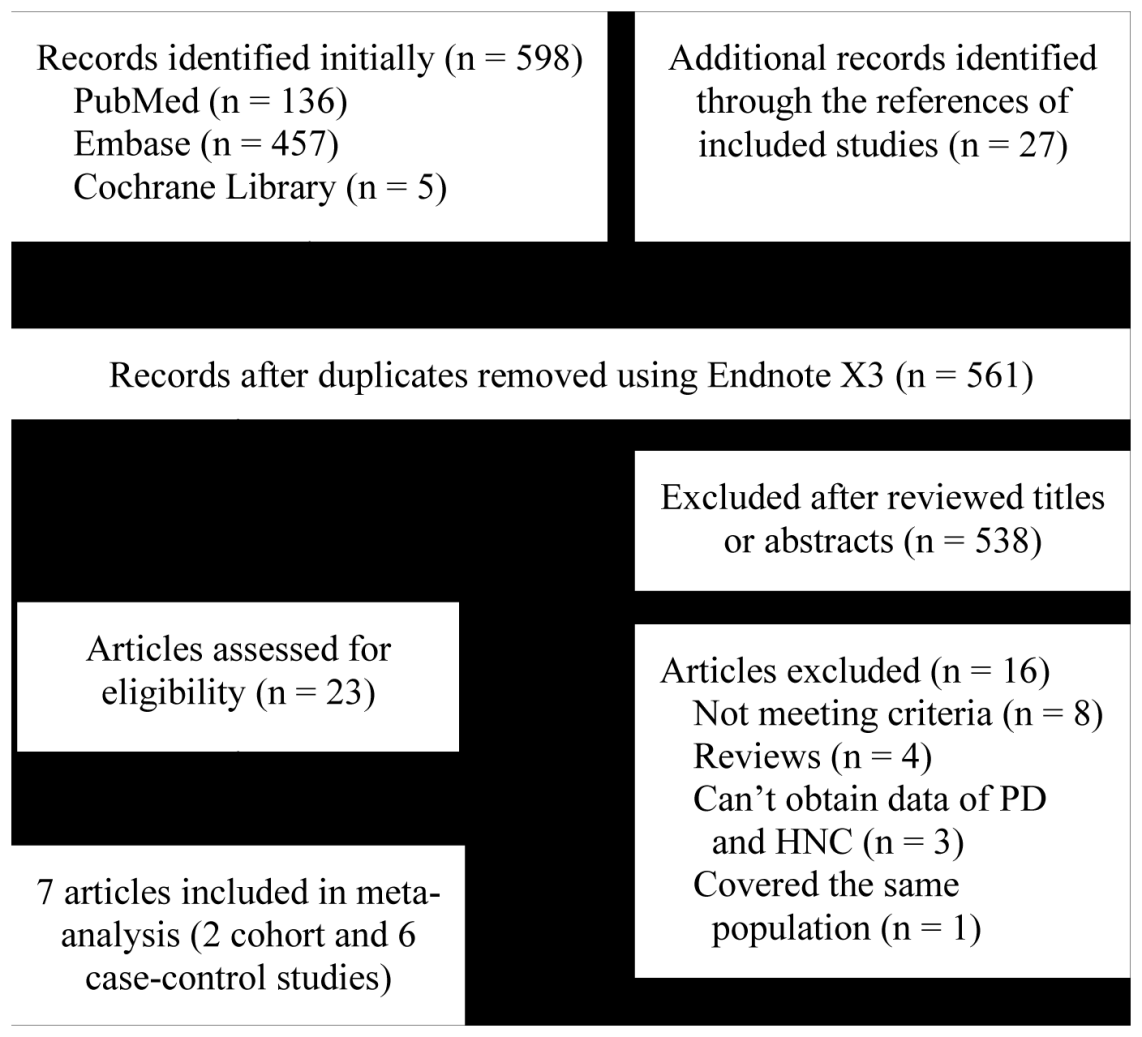
Flow chart from identification of eligible studies to final inclusion. PD, periodontal disease; HNC, head and neck cancer.

Among the 625 records initially searched, seven eligible studies involving two cohort studies [12,15] and six case-control studies [12–18] were included in the meta-analysis. A detailed flowchart of the selection process was shown in Figure 1.

Table 1 shows the major characteristics of the seven studies. The included studies were all published in English and preformed in the following countries (*n*= 8): USA, Brazil, Sweden, Russia, Romania, Poland, Argentina, and Cuba. Among them, six were single -center studies [12,13,15–18], and one contained two multicenter studies [14]. All cases were histologically, pathologically, or cytologically confirmed as HNC. One study was oral tumor (non-specific) [12], one was oropharyngeal cancer [15], two were oral and oropharyngeal SCCs [13,16], and four were HNSCCs (including oral cavity, pharynx, and larynx SCCs) [14,17,18]. One study reported the total number and events of case and control groups [16], three studies only reported adjusted ORs and 95% CIs [12,14,18], one only reported adjusted HR and 95% CI [15], and two reported both crude and adjusted data [13,17]. Table 2 shows the adjusted covariates. PD was defined by alveolar bone loss (ABL) with radiography in 3 studies [13,15,17], by clinical attachment loss (CAL) in one study [12], by community Periodontal Index of Treatment Needs (CPITN) score in one study [16], by tooth mobility in one study [18], and by poor mouth conditions in one study [14].

**Table 1:** Characteristics of included studies in the meta-analysis.

**References**	**Country**	**Study design**	**Sample sizes**	**Age (yrs)**	**Assessment of PD**	**Outcomes**	**Follow-up (yrs)**
Tezal 2005	USA	Cohort	Ca:131	54.6±15.9	CAL	Oral tumor	6
Rosenquist 2005	Sweden	Case-control	Ca:132/Co:320	Ca:33-87/Co:33-89	ABL	OOPSCC	/
de Rezende 2008	Brazil	Case-control	Ca:50/Co:50	>40	CPITN	OOPSCC	/
Tezal 2009	USA	Case-control	Ca:266/Co:207	Ca:56.89±11.73/Co:54.00±15.45	ABL	HNSCC	/
Divaris 2010	USA	Case-control	Ca:1289/Co:1361	Ca:58.9/Co:61.5	Tooth mobility	HNSCC	/
Michaud 2008	USA	Cohort	Ca:118	40-75	ABL	OPC	17.7
Guha E 2007	Europe	Case-control	Ca:792/Co:928	any age	PCM	HNSCC	/
Guha LA 2007	Latin-America	Case-control	Ca:2113/Co:1805	any age	PCM	HNSCC	/

Guha E 2007, the study conducted in Europe, included Russia, Romania, and Poland; Guha LA 2007, the study conducted in Latin-America, included Argentina, Cuba, and Brazil; Ca, cases; Co, controls; CAL, clinical attachment loss; ABL, alveolar bone loss; CPITN, Community Periodontal Index of Treatment Needs; PCM, poor condition of the mouth; OOPSCC, oral and oropharyngeal squamous cell carcinoma; OPC, oropharyngeal cancer; HNSCC, squamous cell carcinoma of the head and neck.

**Table 2:** Adjustments in studies included in this meta-analysis.

**References**	**Adjustment**
Tezal 2005	age, gender, race/ethnicity, education, tobacco, alcohol, occupational hazard, and interaction term tobacco - occupational hazard
Rosenquist 2005	smoking and alcohol
Tezal 2009	age at diagnosis, gender, race/ethnicity, marital status, smoking, alcohol, and missing teeth
Divaris 2010	age, sex, race, education, smoking, alcohol, and fruit and vegetable consumption
Michaud 2008	age, ethnicity, physical activity, history of diabetes, alcohol, smoking, body-mass index, geographical location, height, calcium intake, total calorific intake, red-meat intake, fruit and vegetable intake, and vitamin D score
Guha 2007	age, sex, country, education, smoking, alcohol, and all other oral health variables

### Overall estimates

**Figure 2:**
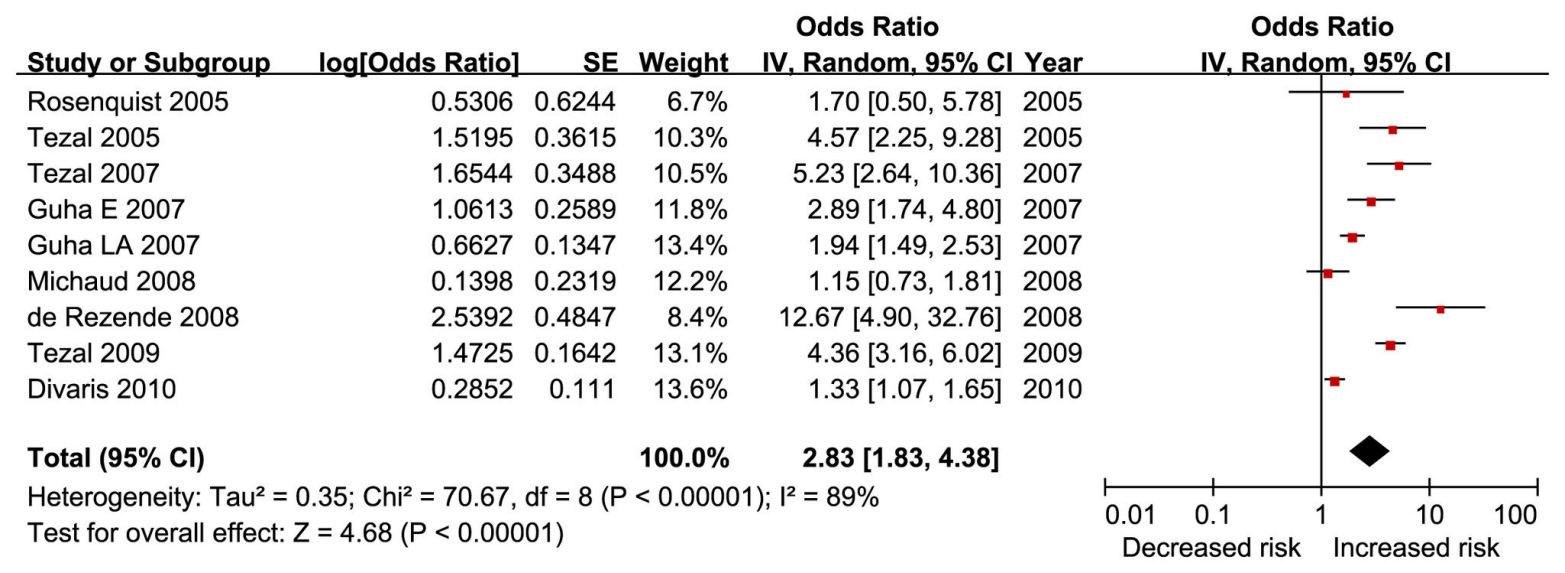
Forest plot of periodontal disease and risk of head and neck cancer, studies are pooled with random-effects model. Guha E 2007, the study conducted in Europe, included Russia, Romania, and Poland; Guha LA 2007, the study conducted in Latin-America, included Argentina, Cuba, and Brazil.

The overall OR estimates for each study were pooled to give a total estimate of risk (Figure 2). Obviously heterogeneity was observed (p < 0.001, I^2^ = 89%), so the result based on the random-effects model showed that exposure to PD can increase the HNC risk 2.63 times (OR = 2.63, 95%CI = 1.68 - 4.14, p < 0.001).

When the study that reported only HR was omitted [15], the result showed that exposure to PD can increase the HNC risk 3.01 times (OR = 3.01, 95%CI = 1.84 - 4.93, p < 0.001), and heterogeneity was also present (p < 0.001, I^2^ = 89%). Thus, the overall result was not changed in the sensitivity analysis.

### Subgroup analysis

Table 3 shows the results of subgroup analysis by adjustment for covariates, study design, PD assessment, tumor site, and ethnicity. All these analyses indicated that PD was a significant risk factor of HNC, except for the cohort design (OR = 2.23, 95% = 0.58 - 8.61, p = 0.24), oral and oropharyngeal SCCs (OR = 4.82, 95%CI = 0.67 - 34.48, p = 0.12), Latin American population (OR = 4.68, 95%CI = 0.75 - 29.33, p = 0.10), and PD assessment by ABL (OR = 1.11, 95%CI = 0.74 - 5.98, p = 0.16). However, when the random-effects model was switched to the fixed effect model for these exceptions, the results were all significant, i.e., for cohort design (OR = 1.72, 95% = 1.17 - 2.52, p = 0.005), oral and oropharyngeal SCCs (OR = 5.95, 95%CI = 2.81 - 12.61, p < 0.001), Latin American population (OR = 2.22, 95%CI = 1.72 - 2.86, p < 0.001), and ABL (OR = 2.73, 95%CI = 2.11 - 3.53, p < 0.001).

**Table 3:** Results of overall and subgroups analyses of pooled ORs and 95% CIs.

**Total and subgroups**	**No. of trails**	**Heterogeneity**		**Model**	**Meta-analysis**		
		**I^2^(%)**	**p**		**ORs**	**95%CIs**	**p**
**Total**	8	89	<0.001	Random	2.63	1.68-4.14	<0.001
**Adjustment for covariates**							
Yes	7	88	<0.001	Random	2.23	1.46-3.43	<0.001
No	1	/	/	Fixed	12.67	4.90-32.76	<0.001
**Study design**							
Cohort	2	90	0.001	Random	2.23	0.58-8.61	0.24
Case-contorl	6	90	<0.001	Random	2.82	1.66-4.78	<0.001
**Assessment of PD**							
ABL	3	88	<0.001	Random	2.11	0.74-5.98	0.16
CAL	1	/	/	Fixed	4.57	2.25-9.28	<0.001
TM	1	/	/	Fixed	1.33	1.07-1.65	0.01
CPITN	1	/	/	Fixed	12.67	4.90-32.76	<0.001
PCM	2	46	0.17	Random	2.23	1.54-3.23	<0.001
**Tumor site**							
Oral cavity	5	90	<0.001	Random	3.08	1.60-3.93	<0.001
Oral and OP	2	85	<0.001	Random	4.82	0.67-34.48	0.12
Larynx	4	52	0.1	Random	1.79	1.34-2.38	<0.001
Pharynx	4	82	0.0007	Random	2.72	1.55-4.77	0.0005
**Ethnicity**							
USA	4	94	<0.001	Random	2.29	1.11-4.76	0.03
Latin-America	2	93	0.0002	Random	4.68	0.75-29.33	0.1
Europe	2	0	0.43	Fixed	2.67	1.67-4.27	<0.001

CAL, clinical attachment loss; ABL, alveolar bone loss; TM, tooth mobility; CPITN, Community Periodontal Index of Treatment Needs; PCM, poor condition of the mouth; OP, oropharynx.

### Publication bias

**Figure 3:**
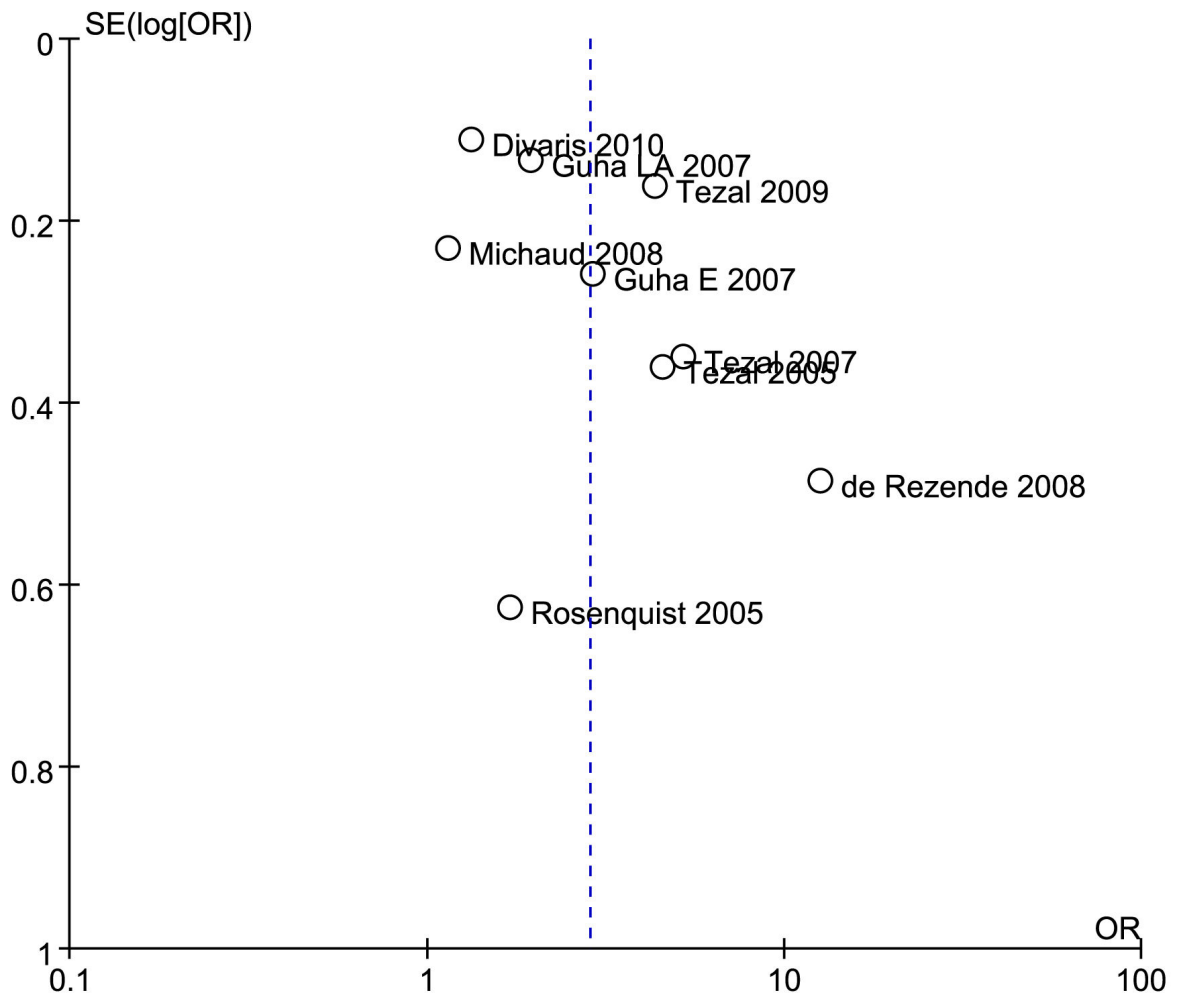
Funnel plot based on overall results of included 2 cohort and 6 case-control studies. Guha E 2007, the study conducted in Europe, included Russia, Romania, and Poland; Guha LA 2007, the study conducted in Latin-America, included Argentina, Cuba, and Brazil.

Figure 3 shows that the funnel plot was not asymmetrical, thereby indicating no significant publication bias.

## Discussion

### Main findings

HNC is known for its rapid clinical progression and poor prognosis, and the survival rates barely improved over the past 40 years. Identifying and preventing the risk factors of HNC are important tasks. Smoking and alcohol are the classical and major risk factors of HNC [3], and successful tobacco and alcohol control programs for reducing the risk of HNC have been developed [1,26]. However, HNC cannot be completely prevented. PD is also considered as an important risk factor of HNC. Accordingly, this study was aimed to estimate the pooled size of PD in HNC risk by a meta-analysis. The pooled effect estimates from seven included papers involving eight studies demonstrated a 2.63 fold (95%CI = 1.68 - 4.14) increased risk of HNC in PD patients compared with PD-free individuals. Sensitivity and subgroup analyses revealed an association between the two diseases when analysis was restricted to adjustment for covariates, study design, PD assessment, tumor site, and ethnicity.

### Sources of heterogeneity

Substantial heterogeneity was observed among the studies because of the differences in population characteristics, study design, PD ascertainment, HNC sites, and adjustment for confounding factors. Our sensitivity analysis suggests that the study of Michaud et al [15] did not contribute to the heterogeneity. Subgroups analysis showed increased heterogeneity in ethnicity and study design groups, that heterogeneity was not caused by ethnicity or study design. However, we cannot exclude them, because the sample sizes were smaller than the overall population. Heterogeneity was decreased in PD ascertainment, HNC sites, and adjustment for confounding factors indicating that they were the factors of heterogeneity. Similar to ethnicity and study design, we cannot exclude the influence of sample sizes. The small number of cases and healthy controls can also increase the possibility of heterogeneity.

### Implications for practice and research

The oral cavity can become a reservoir of respiratory pathogens [27], mainly because of dental plaque and thus contribute to PD [28]. Patients with oral cancer often have poor oral health in general, i.e., they have carious teeth and PD. However, oral health -related variables are also associated with the use of tobacco and alcohol. Thus whether PD is an independent risk factor or only a maker of HNC is unknown. Interestingly, all the six studies [12–15,17,18] with adjustment for covariates including smoking and alcohol showed significantly higher risk (OR = 2.23, 95% = 1.46 - 3.43). This result indicated that PD cannot act as a confounder or an effect modifier for tobacco smoking and alcohol. Therefore, PD was an independent risk factor of HNC. Accordingly, we could deduce that an effective oral hygiene regimen may effectively prevent HNC progression, and an effective PD intervention treatment should be able to improve the prognosis of HNC patients. However, sufficient relevant randomized controlled trials must be conducted to substantiate these findings. If these findings are confirmed, the government medical departments should urgently to increase their budgets for health education and health intervention programs.

Specific oral bacteria have been suggested to be involved in carcinogenesis [29]. Significant differences have been observed when microbial populations in mouth mucosa are compared between healthy and malignant sites. PD is due to oral microorganisms [27]; however, the carcinogenic mechanisms that can explain how PD causes HNC is unclear. Whether PD is only a biomarker for microbe-associated risk of HNC is also undertermined. Therefore, animal experiments should be performed to investigate the true mechanism between PD and HNC, particularly to determine the mechanism and identify the pathogens involved both in PD and HNC. The results can give implications for clinical practice and support the link between the two diseases. If such a link is proven, the related organizations such as the International Agency for Research on Cancer and the American Dental Association should list PD as a risk factor of HNC and formulate policies to promote oral health care.

The included studies were performed in non-Asian countries, and most Asian countries are developing countries. Given that access to dental care is limited in these countries, we predict that the prevalence/incidence of HNC in these countries will higher than the developed countries (where access to dental care is better). Therefore, we suggest that the developing countries carry out relevant studies to provide evidence and verify this prediction. In addition, the tools for PD assessment varied in the included studies. In future studies, researchers should choose a validated and optimal measurement (e.g., CPITN [30]) of PD, to decrease heterogeneity and increase accuracy.

### Limitations of study

This study is the first meta-analysis investigating the association between PD and HNC and has some limitations. First, our meta-analysis was based on observational studies, and all included studies with adjustment groups have controlled the classical and major risk factors (smoking and alcohol) even though this confounding factor is difficult to control in epidemiological studies [31].Thus, whether PD is a risk factor or only a significantly biomarker for oral microorganisms. Second, we performed a thorough literature review and examination of reference lists in published articles to identify all published studies, and the funnel plot indicated that no obviously publication bias existed; however, the plot was not very symmetrical and the number of studies for our meta-analysis is not large. Third, heterogeneity was detected in our meta-analysis. Heterogeneity between studies should not be ignored even if it is highly common in the meta-analysis of genetic association studies. Moreover, we performed subgroup analyses to verify the data, but heterogeneity was also observed. Fourth, the sample sizes in many studies were relatively small. Compared with the studies having a large sample size, studies with a small sample size may overestimate the true association. A large sample study with either finding may better reflect a true association because of its sufficient statistical power. Fifth, the assessment tools for identifying PD were different, and relevant data of developing countries based on the currently included studies were not obtained. Overall, these aforementioned limitations may affect our final conclusions.

## Conclusions

The meta- analysis of all published epidemiological studies on PD and HNC revealed that PD significantly increases the risk of HNC and that the increase is probably independent of conventional HNC risk factors.

## Supporting Information

Checklist S1 **PRISMA Checklist.**
(DOC)
